# Role of posttranslational modifications in memory and cognitive impairments caused by neonatal sevoflurane exposure

**DOI:** 10.3389/fphar.2023.1113345

**Published:** 2023-03-13

**Authors:** Yongliang Jiang, Yue Zhou, Siwen Tan, Chongxi Xu, Junpeng Ma

**Affiliations:** ^1^ Department of Neurosurgery, West China Hospital of Sichuan University, Chengdu, China; ^2^ Department of Pharmacy, Xindu District People’s Hospital of Chengdu, Chengdu, China; ^3^ Outpatient Department, West China Hospital of Sichuan University, Chengdu, China

**Keywords:** posttranslational modifications, neonatal, sevoflurane, memory and cognitive impairments, long term

## Abstract

With the advancement of technology, increasingly many newborns are receiving general anesthesia at a young age for surgery, other interventions, or clinical assessment. Anesthetics cause neurotoxicity and apoptosis of nerve cells, leading to memory and cognitive impairments. The most frequently used anesthetic in infants is sevoflurane; however, it has the potential to be neurotoxic. A single, short bout of sevoflurane exposure has little impact on cognitive function, but prolonged or recurrent exposure to general anesthetics can impair memory and cognitive function. However, the mechanisms underlying this association remain unknown. Posttranslational modifications (PTMs), which can be described roughly as the regulation of gene expression, protein activity, and protein function, have sparked enormous interest in neuroscience. Posttranslational modifications are a critical mechanism mediating anesthesia-induced long-term modifications in gene transcription and protein functional deficits in memory and cognition in children, according to a growing body of studies in recent years. Based on these recent findings, our paper reviews the effects of sevoflurane on memory loss and cognitive impairment, discusses how posttranslational modifications mechanisms can contribute to sevoflurane-induced neurotoxicity, and provides new insights into the prevention of sevoflurane-induced memory and cognitive impairments.

## Introduction

The standard of care for a wide range of procedures and surgeries on newborns is general anesthesia. General anesthetics are administered to millions of young children each year to facilitate surgical procedures and examinations (DeFrances et al., 2007). Each year in the United States, nearly 650,000 children under three years of age will be exposed to general anesthesia (Warner et al., 2018). Although general anesthesia is typically thought of as safe and reversible in adults, there has recently been public concern that the high rates of synaptogenesis, neurogenesis, and neuronal maturation in the developing brain may increase the danger of adverse long-term effects of anesthetic exposure (Servick, 2014). In particular, recent studies have suggested that general anesthesia given in the earliest stages of life may result in aberrant brain development (Sanders et al., 2013). For instance, approximately 12% of patients who appear to have normal cognitive function prior to anesthesia and non-cardiac surgery exhibit signs of cognitive dysfunction after surgery (Needham et al., 2017). Since this discovery, preclinical investigations on how anesthetics affect the developing brain have grown significantly (Lin et al., 2017; Jevtovic-Todorovic, 2018). For example, sevoflurane exposure at critical stages of development may result in neuronal apoptosis and cognitive impairments, according to experimental research on animals (Sun et al., 2021).

Sevoflurane, which was first used in clinical practice in the 1990s, has become one of the most often used inhalational anesthetics in pediatric operations because it offers better hemodynamic stability, speedier recovery, and less respiratory irritation than other anesthetics (Brioni et al., 2017). However, there is conclusive evidence that repeated exposure to sevoflurane-based anesthetics in both humans and animals can cause neuropathological alterations in the brain and lead to long-term cognitive impairment (Tang et al., 2020). Numerous preclinical studies have demonstrated that prolonged or recurrent exposure to sevoflurane alone during the first postnatal days may result in behavioral abnormalities and learning and memory impairments later in life in rodents (Vutskits and Xie, 2016). Numerous efforts have been made to solve this problem, and several advances have been made recently. However, uncertainty exists regarding the processes underlying the long-term effects of neonatal sevoflurane exposure on brain development.

Through covalent or enzymatic alterations, posttranslational modifications (PTMs) transforms newly transcribed proteins into mature proteins (Gupta et al., 2021). These alterations range from the covalent insertion of particular chemical groups, lipids, carbohydrates, whole proteins, and even amino acid side chains to the enzymatic breakdown of peptide bonds (Gupta et al., 2021). Since these chemical changes following polypeptide chain formation broaden the range of amino acid structures and characteristics, protein structure and function become more varied. PTMs controls protein activity, localization, and interactions with other molecules and can occur at any point (Knorre et al., 2009). Numerous studies have shown how various aberrant PTMs are involved in the etiology of dysfunctional brain function and neurodegenerative disorders (Varland et al., 2015; Gupta et al., 2021). In addition, numerous studies have demonstrated the functions of PTMs in memory formation or memory loss (Zovkic et al., 2013; Kim and Kaang, 2017), and more recently, some scientists have concentrated on the impacts of PTMs on neurotoxicity induced by general anesthesia in newborns (Wu et al., 2019; Yu et al., 2022). For example, more evidence has demonstrated that irregular PTMs affect neuron activity and function, which induces the progression of sevoflurane-induced cognitive impairment (Dong et al., 2021; Zuo et al., 2021; Wang et al., 2022). These data suggest that PTMs may be beneficial for treating cognitive impairment after sevoflurane exposure by altering neuronal activity and function.

Regarding sevoflurane, there is no doubt that prolonged exposure is harmful to brain development in both animal studies and clinical studies. Children’s memory and cognitive abilities are affected by sevoflurane-induced neurotoxicity, although the underlying mechanisms of this effect and the related processes are still not completely clear (Wu and Zhao, 2018). To date, there is no effective medicine that can block the progression of cognitive impairment. PTMs dysregulation is now receiving much attention for playing a crucial role in anesthesia-induced cognitive impairment at the beginning of brain development, which is a novel mechanism that has not been fully illustrated in cognitive impairment after sevoflurane exposure (Wang et al., 2021). Thus, in this review, we discuss several PTMs dysregulation mechanisms in histones and non-histone proteins that have been linked to and may be responsible for sevoflurane-induced memory and cognitive impairments (Table 1). We hope that a new mechanism of PTMs might be a viable therapeutic target for either partial or full treatment of sevoflurane-induced cognitive dysfunction.

**TABLE 1:** Summary of previously identified PTM of protein involved in sevoflurane-induced neurotoxicity.

References	Animals	Sevoflurane administration protocol	Acetylation	PTMs phosphorylation	Methylation
Chai et al. (2022)	Mice	3% sevoflurane for 2 h daily for 3 consecutive days	H3K14, H3K18 H4K5, H4K12		
Jia et al. (2016)	Rats	3% sevoflurane for 2 h daily for 3 consecutive days	H3K9, H3K14 H4K12		
Yu et al. (2022)	Mice	3% sevoflurane plus 60% oxygen twice daily for 3 days		Tau-S202, Tau-T205 Tau-S396, Tau-S404	
Tao et al. (2014)	Mice	3% sevoflurane for 2 h daily for 3 consecutive days		Tau-S202, Tau-T205	
Le Freche et al. (2012)	Mice	1.5 or 2.5% sevoflurane repeated (5 exposures of 1 h every month)		Tau-S396, Tau-S404 Tau-T181	
Yu et al. (2020)	Mice	3% sevoflurane for 2 h daily for 3 consecutive days		Tau-S202, Tau-T205 Tau-S356	
Dong et al. (2021)	Mice	Primary neurons in 3% sevoflurane for 2 h daily for 3 consecutive days		Tau-S202, Tau-T205	
Wu et al. (2019)	Mice	1.4% isoflurane for 2 h (speculation sevoflurane has a similar function)			H3K9

## Posttranslational modifications of histones

**FIGURE 1:**
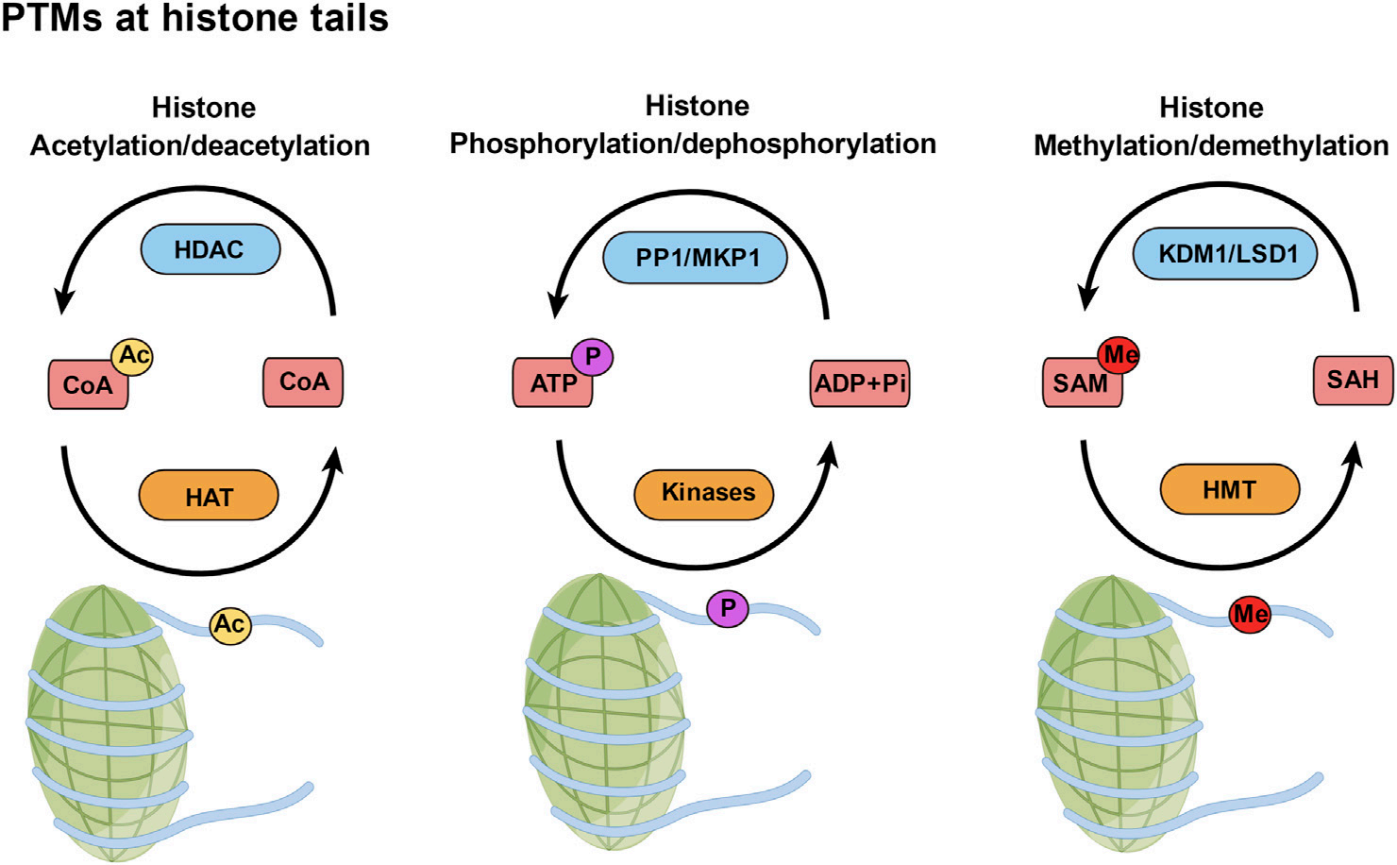
Posttranslational modifications (PTMs) at histone tails. PTMs of histones, such as acetylation, phosphorylation, and methylation, are added to those amino acids by writer enzymes (orange) and removed by eraser enzymes (blue). Ac, acetyl; P, phosphate; Me, methyl.

Chromatin geometry and gene expression depend on posttranslational histone modifications (Inaba et al., 2015) (Figure 1). The positive charges of unmodified histone proteins encourage a close association with the negative charges of DNA, which puts chromatin in an unfavorable state for transcription. However, the acetylation, phosphorylation, and methylation of histones alter their charges and binding characteristics (Doerks et al., 2002). Since acetylated histones serve as binding sites for transcriptional machinery, they are frequently linked to transcriptional activation (Wu and Zhao, 2018). Histone methylation can facilitate both transcriptional activation and repression, but histone phosphorylation is linked to transcriptional activity (Maity et al., 2021; Ma et al., 2022). By activating transcriptional machinery, these coupled PTMs of histone proteins act as a “histone code” and control gene expression (Strahl and Allis, 2000).

### Histone acetylation

Histone acetylation is one of the most researched mechanisms among the numerous forms of histone modifications, and studies have demonstrated the significance of these alterations for brain development and function (Keverne, 2014; Sen, 2015). This procedure involves attaching an acetyl group to a lysine at the nucleosome’s N-terminal tail, which is the basic unit of DNA packing in eukaryotic cells. Histone acetyltransferases (HATs) and histone deacetylases (HDACs) are enzymes that catalyze the acetylation of histones. By removing acetyl groups from histone tails, HDACs reverse the activity of HATs and inhibit transcription (Berndsen and Denu, 2008; Wu and Zhao, 2018).

It is commonly believed that histone acetylation is advantageous for memory and cognition, since it is frequently linked to an increase in the expression of many genes that play significant roles in synaptic plasticity, learning, and memory in the brain (Kim and Kaang, 2017). Recent studies have shown long-term developmental cognition deficits and changes in histone acetylation in rodents exposed to anesthetics during the prenatal or neonatal phase. For instance, adult mice that were exposed to sevoflurane during the neonatal period had higher hippocampal levels of HDAC3 and HDAC8 but not HDAC1 and HDAC2. Additionally, rats exposed to sevoflurane had lower levels of acetylated H3K14/18 and H4K5/12 in the hippocampus (Chai et al., 2022) and lower levels of many genes involved in neurodevelopment and neuroplasticity, such as c-Fos, brain-derived neurotrophic factor (BDNF), and postsynaptic density protein 95 (PSD-95) (Wu and Zhao, 2018). It is important to note that sevoflurane exposure is linked to alterations in specific regions of the brain. For example, H3K9/14 and H4K12 acetylation were decreased in the hippocampal CA1 region, and H3K14 acetylation was decreased in both the CA1 and dentate gyrus (DG) regions of the hippocampus (Jia et al., 2016). While some studies have shown that histone hypoacetylation is controlled by an increase in HDACs, some studies have demonstrated opposing results. These studies have indicated that this process involves a decrease in histone acetylation and is a key factor in the memory and cognitive impairments caused by neonatal anesthesia. Through the use of the HDAC inhibitors trichostatin A (TSA) and sodium butyrate, researchers were able to successfully reduce the neurocognitive impairment induced by general anesthesia as well as the dysregulation of histone acetylation in the hippocampus (Zhong et al., 2015; Jia et al., 2016).

### Histone phosphorylation

The most frequent posttranslational change in eukaryotes is protein phosphorylation. The creation of phosphodiester bonds can occur on the side chains of serine, threonine and tyrosine (often referred to as “residues”). Numerous protein kinases, protein phosphatases, and phosphorylated proteins are found in neurons, and many of these are crucial for controlling neuronal morphology and for a variety of cell processes, including membrane excitability, secretory procedures, cytoskeletal organization, and cellular metabolism (Li et al., 2021).

Histone phosphorylation is another PTM that offers a distinctive epigenetic signature to control chromatin dynamics (Graff and Mansuy, 2008). The “writer” enzymes responsible for histone phosphorylation are kinases, which mediate the transfer of a phosphate moiety from the high-energy molecule ATP. In contrast, the “eraser” enzymes that remove these phosphate groups are protein phosphatase 1 (PP1) and MAPK phosphatase 1 (MKP1) (Torres-Perez et al., 2021). Due to its link to chromosome condensation during mitosis, histone H3 phosphorylation has attracted considerable attention (Bradbury et al., 1973; Gurley et al., 1974; Gurley et al., 1978). Interestingly, the activation of mitogenic signaling pathways is the origin of H3 phosphorylation (Mahadevan et al., 1991). For example, ribosomal protein S6 kinase 2 (RSK2) is downstream of several other kinases, including extracellular signal-regulated kinase (ERK), aurora kinase family member increase in ploidy 1 (IPL1), and mitogen- and stress-activated protein kinase 1 (MSK1), and RSK2 mediates the phosphorylation of histone H3 on the serine 10 residue (Sassone-Corsi et al., 1999; Thomson et al., 1999; Hsu et al., 2000). In addition, H3 phosphorylation controls the binding of the transcriptional apparatus to chromatin molecules, which modulates key biological processes in conjunction with other histone modifications.

MSK1 is crucial in activating the histone phosphorylation process. This is supported by the fact that long-term spatial and contextual fear memory development is impaired by MSK1 germline deletion (Chwang et al., 2007). In addition to MSK1, the IB kinase (IKK) complex also controls histone phosphorylation in the hippocampal nucleus and memory formation (Lubin and Sweatt, 2007). The above studies show that histone phosphorylation is crucial for the development of memories. However, studies investigating the involvement of histone hypophosphorylation in sevoflurane-induced cognitive impairment have not been reported to date. Perhaps this will be investigated further and tested in the near future.

### Histone methylation

Histone methylation is another mechanism that controls chromatin structure; methyl groups can be added by histone methyltransferases (HMTs) or removed by FAD-dependent amine oxidase (KDM1 or LSD1) (Torres-Perez et al., 2021). In addition, histone methylation can cause transcriptional activation, even though methylation is typically thought of as a signal for transcriptional repression. When histone H3 is methylated at lysine 9 (H3K9), it is related to transcriptional repression. However, when it is methylated at lysine 4 (H3K4), it is related to transcriptional activation (Vermeulen et al., 2007). The role of histone methylation in long-term memory storage has been the subject of recent research (Gupta et al., 2010). Researchers have found that hippocampal H3K4 is trimethylated and hippocampal H3K9 is dimethylated throughout the process of memory formation, indicating that gene expression alterations exist during this process (Gupta et al., 2010). After surgery under general anesthesia for 24 h, mice showed a substantial increase in H3K9me3 expression and its binding to the BDNF exon IV promoter, while BDNF expression was downregulated (Wu et al., 2019). Although middle-aged mice were used as the study model, these findings enhance our understanding of neonatal anesthetic neurotoxicity, especially in light of the bidirectional regulation of this change and the paucity of in-depth research.

HMTs and histone demethylases are enzymes that regulate histone methylation. SET-domain HMTs, non-SET-domain DOT1/DOT1L methyltransferases, and PRMT1 arginine methyltransferases are the three enzyme families that compose the HMTs (Jarome and Lubin, 2013). Arginine and lysine residues may undergo histone methylation. Lysine methylation on histones H3 and H4 is much more extensively studied. Histone methylation is improved in the hippocampus after treatment with sodium butyrate, an HDAC inhibitor. This implies a functional connection between histone methylation and acetylation during the consolidation of terror memories (Gupta et al., 2010). Additionally, research has shown that mice lacking the histone methyltransferase myeloid/lymphoid or mixed-lineage leukemia 2 (MLL2/KMT2B) gene in adult excitatory neurons display memory deficit tasks that depend on the hippocampus (Kerimoglu et al., 2013). By correcting the transcriptional imbalance of memory-related genes, it is feasible to use histone methylation as a therapeutic target for sevoflurane-induced cognitive impairment.

## Posttranslational modifications of nonhistone proteins

Amyloid-β protein (Aβ), the main component of senile plaques in AD patients, was initially found in meningovascular amyloid deposits in AD and Down syndrome patients (Glenner and Wong, 1984; Selkoe et al., 1988; Goate et al., 1991). Aβ is created from its massive precursor protein, amyloid precursor protein (APP), by two proteases, β-secretase (β-site APP-cleaving enzyme, or BACE) and γ-secretase, which sequentially cleave the proteins. Additionally, another secretase, α-secretase, can likewise cleave APP at a site close to the transmembrane domain and in the middle of the Aβ region (Xie and Xu, 2013).

Senile plaques and Aβ peptide deposits are the initial neuropathological signs of AD (Martin et al., 2011). Similar to how the nitration of tyrosine 10 by nitric oxide (NO) can cause Aβ aggregation, the glycosylation of tyrosine 10 alters γ-secretase cleavage because of the proximity of PTMs to the transmembrane domain (Warmack et al., 2019). *In vitro* aggregation and β-sheet formation can be increased as a result of the polyglutamylation of glutamate 11. Additionally, plaques have higher levels of racemization of aspartic acid 1, while serine 26 exhibits a greater propensity to produce fibrils (Warmack et al., 2019). This is similar to how APP processing and trafficking are controlled by O-linked N-acetylglucosaminylation (O-GlcNAcylation) at threonine 576, which increases the number of toxic aggregates produced by APP (Chun et al., 2017).

Previous research revealed that exposing H4 human neuroglioma cells to 4.1% sevoflurane for 6 h caused caspase activation and death, increased BACE levels, decreased APP 99-residue membrane-associated C-terminus fragment (APP-C99) and 83-residue membrane-associated C-terminus fragment (APP-C83) levels, and eventually increased Aβ levels (Dong et al., 2009). Additionally, the caspase inhibitor Z-VAD reduced the effects of sevoflurane on the ability of cells to process APP and accumulate Aβ (Dong et al., 2009). Finally, in H4 human neuroglioma cells, sevoflurane enhanced caspase-3 activation, and the reduction in Aβ generation by the γ-secretase inhibitor L-685,458 decreased sevoflurane-induced caspase-3 activation (Dong et al., 2009). These results indicate that sevoflurane can activate caspases and cause apoptosis. This increases BACE levels, promotes PTM of APP, and increases Aβ production. Aβ then enhances the apoptosis and caspase activation caused by sevoflurane. Another study examined the effects of sevoflurane in young mice and discovered that the brains of six-day-old mice underwent caspase activation and apoptosis following anesthesia with 3% or 2.1% sevoflurane for 6 hours (Lu et al., 2010). Additionally, 3% sevoflurane anesthesia for 6 h caused more caspase activation in the brain tissues of AD transgenic mice than in those of wild-type mice. Sevoflurane anesthesia increased the levels of Aβ in the brain tissues of six-day-old mice (Lu et al., 2010). These findings demonstrate that sevoflurane can increase brain Aβ levels, even in newborn mice. Additionally, repeated PTMs can cause high Aβ levels, resulting in the development of Aβ protofibrils. This may exacerbate the neurotoxicity caused by sevoflurane in the developing brain (Lu et al., 2010; Xie and Xu, 2013).

### Tau phosphorylation

Phosphorylation plays a crucial role in regulating the physiological functions of Tau, including its binding to microtubules, and therefore regulates the stabilization and assembly of microtubules themselves. Hyperphosphorylation of Tau may induce brain pathology and is one of the earliest events in the development of AD, playing an important role in the impairment of neuronal function, memory, and cognition. Tau phosphorylation provides a new perspective on sevoflurane-induced cognitive dysfunction in neonatal mice (Yu et al., 2022). Sevoflurane-induced cognitive impairment in neonatal mice is altered by Tau phosphorylation.

Tau protein was first identified in 1975 as a microtubule-associated protein (Weingarten et al., 1975) that is restricted to the axonal compartment of neurons in the healthy brain (Binder et al., 1985). Tau proteins are a family of microtubule-associated proteins that play a role in microtubule assembly, support axonal integrity in mature neurons (Buée et al., 2000; Sergeant et al., 2008), and perform functions at the dendrites in neurons (Ittner et al., 2010; Sultan et al., 2011). The main components of the intraneuronal paired helical filaments (PHFs) seen in AD are hyperphosphorylated and abnormally phosphorylated forms of Tau. They have also been observed in Tauopathies, which are other neurodegenerative diseases featuring filaments of a similar type (Buée et al., 2000; Buee et al., 2010; Iqbal et al., 2010). Excessive Tau phosphorylation encourages the creation of intractable Tau clumps. Once the aggregate has been produced, it can leave the original cell, come into contact with a connected cell, and then enter the cell to produce more aggregation by altering the conformation of the template (Holmes and Diamond, 2014). In addition, Tau phosphorylation can impair cognitive performance in mice (Faraco et al., 2019).

**FIGURE 2:**
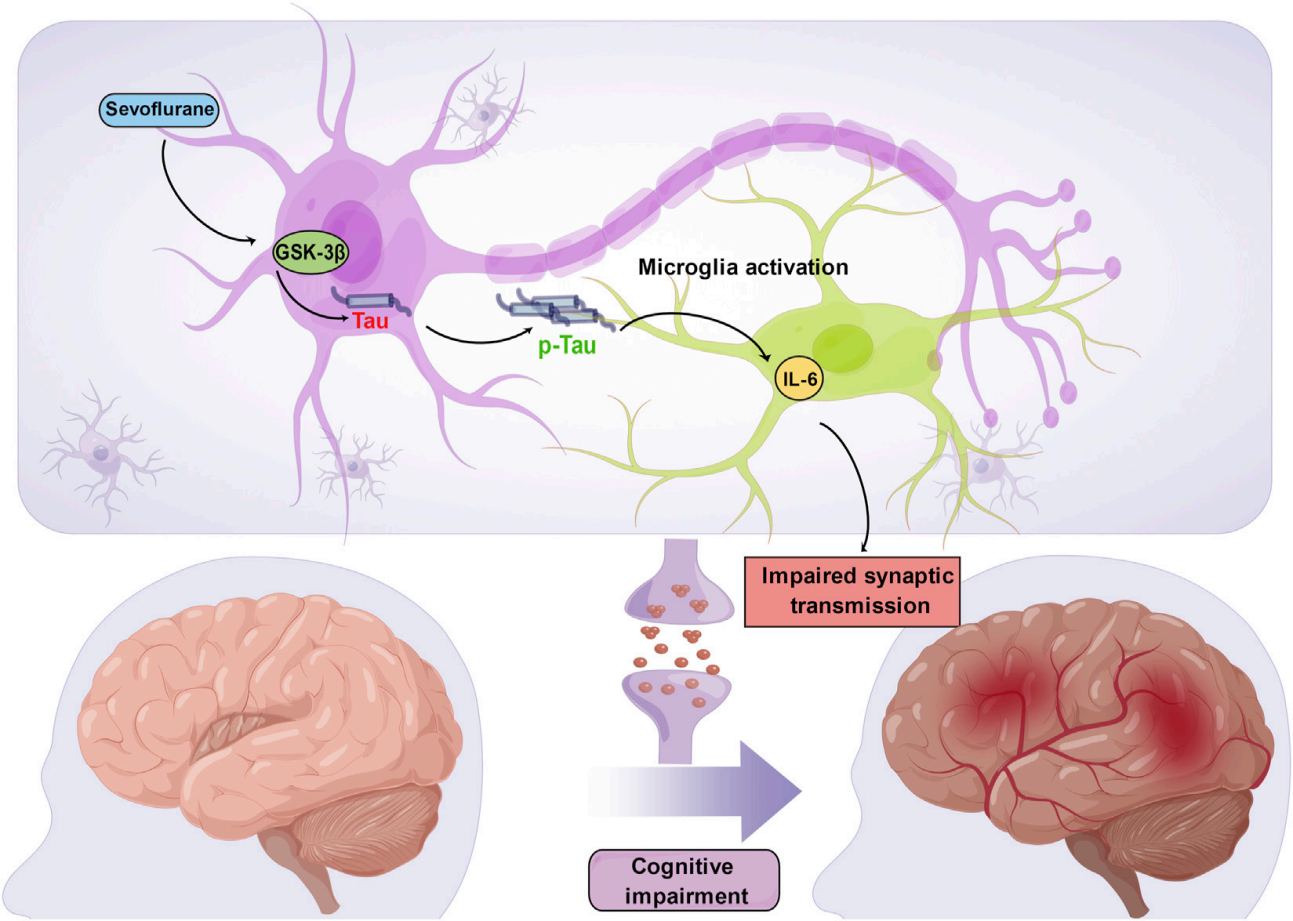
Sevoflurane induces Tau phosphorylation in the developing brain. Sevoflurane increases Tau phosphorylation *via* GSK-3β activation, which induces extracellular vesicle-associated trafficking of hyperphosphorylated Tau from neurons to microglia, triggers microglial activation and produces a large amount of IL-6. High levels of hippocampal IL-6 compromise synaptic transmission and further lead to cognitive impairment.

In mice at postnatal day 6, multiple exposures, but not a single exposure, to 3% sevoflurane for 2 hours daily led to Tau phosphorylation *via* GSK-3β activation. This phosphorylation increased IL-6 levels and decreased PSD-95 levels in the hippocampus, leading to cognitive impairment (Tao et al., 2014). Tau may play a key role in sevoflurane-induced neuroinflammation and synaptic impairments in mice since these side effects of the drug did not manifest in Tau knockout (Tau KO) mice. Because of reduced mitochondrial performance in newborn mice, ATP is decreased and NUAK family kinase 1 (NUAK1) is increased in the brain. According to Lasagna-Reeves et al., increasing NUAK1 may cause Tau phosphorylation at serine 356 and prevent Tau degradation (Lasagna-Reeves et al., 2016), causing more Tau to accumulate in the brain tissues of neonatal mice than in those of adult mice (Yu et al., 2020). Therefore, neonatal mice are more vulnerable than adult mice to the onset of Tau phosphorylation caused by sevoflurane anesthesia (Yu et al., 2022). Acute 1.5% sevoflurane exposure resulted in considerable, dose-dependent, and reversible hyperphosphorylation of Tau in the hippocampus of 5- to 6-month-old mice (Le Freche et al., 2012). Furthermore, previous research demonstrated that repeated exposure to 2.5% sevoflurane under normothermic conditions causes Tau to be persistently hyperphosphorylated at the Ser396/Ser404 and Thr181 phosphoepitopes (Le Freche et al., 2012). The Morris water maze (MWM) test was used to measure subsequent substantial deficits in spatial learning and memory in mice (Le Freche et al., 2012). These earlier investigations highlight a potential mechanism through which anesthetics may impair cognition and increase the risk of AD because hyperphosphorylated Tau is a prominent component of neurofibrillary lesions (Run et al., 2009). However, a direct link between the accumulation of phosphorylated Tau and cognitive impairment needs to be established. IL-6 production and cognitive dysfunction were shown to be the result of the ability of sevoflurane to cause Tau phosphorylation and the extracellular vesicle-associated trafficking of Tau from neurons to microglia (Dong et al., 2021) (Figure 2). As a result, these recent discoveries suggest that Tau phosphorylation may be a factor in sevoflurane-induced memory and cognitive impairments. Future research is undoubtedly needed to better understand how phosphorylated Tau contributes to anesthesia-induced memory and cognitive impairments.

Sex-dependent anesthetic-induced neurotoxicity has been reported in the developing brain (Lee et al., 2014; Ju et al., 2018). The role of sex hormones in these effects has been specifically determined (Yang et al., 2021; Jevtovic-Todorovic, 2022). For example, neonatal male mice had lower levels of brain testosterone and were more vulnerable to the neurotoxic effects of sevoflurane. Treatment with testosterone before sevoflurane anesthesia attenuated sevoflurane-induced Tau phosphorylation and cognitive impairment by decreasing the binding and interaction between Tau and GSK-3β, a kinase that phosphorylates Tau in neonatal male mice (Yang et al., 2021). These results suggest possible protective effects of testosterone on anesthetic-induced neurotoxicity. However, it is important to note that Tau levels in the hippocampus and cortex are higher in neonatal mice than in adult mice, which could account for the increased Tau phosphorylation in neonatal mice after sevoflurane anesthesia (Yu et al., 2020). Testosterone enhanced mitochondrial function in the rat brain by increasing mitochondrial reduced nicotinamide adenine dinucleotide-ubiquinone oxidoreductase chain 1 protein levels and mitigating oxidative damage (Yan et al., 2017). Thus, future work should determine whether neonatal male mice accumulate more Tau and phosphorylated Tau in the brain attributable to impaired mitochondrial function resulting from lower testosterone levels.

We should note that a single exposure of neonatal rats to sevoflurane led to significant behavioral abnormalities not only in exposed rats in adulthood but also in their adult male offspring that were never exposed to sevoflurane (Ju et al., 2018). In addition, anesthetic-induced abnormalities are greater in male animals (Lee et al., 2014; Ju et al., 2018). Male progeny, but not female progeny, of sevoflurane-exposed parents showed abnormalities in behavioral testing and expression of the KCC2 cotransporter, and the male F1 rats of two exposed parents showed impaired spatial memory and KCC2 expression (Ju et al., 2018). These results suggest that there could be sex-dependent differences in sevoflurane-induced neurotoxicity (Chastain-Potts et al., 2020). The neurobehavioral abnormalities induced by neonatal exposure to the general anesthetic sevoflurane can be transmitted to the next-generation in a complex, sex-specific manner through epigenetic mechanisms (Ju et al., 2018). The underlying mechanisms of sevoflurane-induced sex-specific effects across two generations are exciting and challenging topics for future studies. Researchers should make further efforts to elucidate how closely the observed changes in protein PTMs expression relate to sex-specific effects of sevoflurane.

## Conclusion

Due to its distinct pharmacological characteristics, sevoflurane has become the most widely used anesthetic. However, there is mounting evidence suggesting that sevoflurane causes impairments in long-term memory and cognitive function. Although sevoflurane-induced memory and cognitive impairments have been extensively studied, there is currently no effective treatment available. However, PTMs intervention may be a promising prospective targeted treatment to reduce the neurotoxic effects of anesthetics on developing brains, given the efficacy of therapeutic intervention utilizing HDAC inhibitors. In addition, we believe that Tau phosphorylation deserves further attention in the future. Advanced technologies should be applied in future studies of Tau phosphorylation or other protein PTM mechanisms in sevoflurane-induced developmental neurotoxicity. We also note that future studies should focus on elucidating the etiology of sevoflurane-induced memory and cognitive impairments and comprehensively understanding the importance of PTMs in this field to create a more potent combination therapy.
